# Parametric study of glycerol and contaminants removal from biodiesel through solvent-aided crystallization

**DOI:** 10.1186/s40643-021-00409-y

**Published:** 2021-06-29

**Authors:** Mohd. Afnan Ahmad, Arun Letchumanan, Shafirah Samsuri, Wan Nur Athirah Mazli, Juniza Md Saad

**Affiliations:** 1 Chemical Engineering Department, Universiti Teknologi PETRONAS, 32610 Seri Iskandar, Perak, Malaysia; 2 HICoE Centre for Biofuel and Biochemical Research (CBBR), Universiti Teknologi PETRONAS, 32610 Seri Iskandar, Perak, Malaysia; 3 Department of Science and Technology, Faculty of Humanities, Management and Science, Universiti Putra Malaysia, Nyabau Road, 97008 Bintulu, Sarawak Malaysia

**Keywords:** Biodiesel purification, Solvent-aided crystallization, Glycerol, Fatty acid methyl esters

## Abstract

At present, biodiesel is known as an alternative fuel globally. It is also known that the purification of biodiesel before consumption is mandatory to comply with international standards. Commonly, purification using water washing generates a massive amount of wastewater with a high content of organic compounds that can harm the environment. Therefore, this study applied and tested a waterless method, i.e., the solvent-aided crystallization (SAC), to remove glycerol and other traces of impurities in the crude biodiesel. The parameters of coolant temperature, crystallization time, and stirring rate on the SAC system were investigated. It was discovered that with 14 °C coolant temperature, 300 RPM and higher cooling time result in the highest percentage of FAME up to 99.54%, which indicates that contaminants' presence is limited in the purified biodiesel. The use of 1-butanol as the solvent for crystallization process remarkably enhanced the separation and improved the higher biodiesel quality.

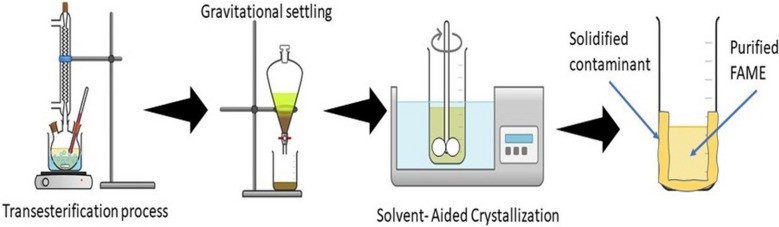

## Introduction

The rise in the economy concerning transportation as well as environmental awareness leads to the use of petroleum-derived fossil fuels. The term biodiesel is referred to as renewable and biodegradable fuel that functions as an alternative in traditional engines (Tyson and McCormick 2006). Vegetable oil, animal fats, and waste cooking oil can be used as feedstock in producing biodiesel such as soybean oil (55%), canola oil (10%), corn oil (12%), recycled used cooking oils, and yellow greases (13%) in the United States in 2016 (Ambat et al. 2018). Oils from rapeseed, sunflower, palm kernel, and animal fats such as beef tallow and pork lard are also considered significant sources to produce biodiesel. To reserve the decreasing of traditional world fuel and promote sustainability to avoid environmental effects, biodiesel is preferred, and it has served as a prominent alternative source of fuel. Compared to petroleum fuel, the use of biodiesel also reduces the greenhouse effects, which can minimize the threat of global warming. Other than that, gasses emitted from biodiesel's combustion have low carbon monoxide content, contributing to the controlled carbon dioxide emissions and air pollution (Mohammed and Bandari 2017).

The process of transesterification is one of the simplest and quickest ways of producing biodiesel. The procedure still induces glycerol as a by-product. Unreacted methanol, residual catalyst, soap, and water are also present in production. After the reaction, glycerol and other by-products are removed from the reaction mixture by a simple gravitational settling process, and the FAME phase washed to remove impurities. However, biodiesel cannot be used directly without purification, which can cause engine knocking or choking on the injector. In addition, the glycerol may accumulate around the injector valve heads and affect engine performance (Saleh et al. 2010; Atadashi et al. 2011). In the American Society for Testing and Materials (ASTM D6751), the removal of glycerol and other by-products from the fatty acid methyl ester (FAME) process is mandatory because the purity of the final FAME product is highly stressed. Despite the fact that biodiesel is the only substitute form of fuel that has been recognized in the testing requirements of the 1990 Clean Air Act Amendment and legally registered as a legal motor fuel with the Environmental Protection Agency, ASTM D6751, and EN 14,214 standards ensure the presence of impurities is limited in the FAME layer of biodiesel (Shah and Porter 2014).

Conventionally, biodiesel is purified using water washing, acid washing, and washing with ether and absorbents (Bateni et al. 2017; Berrios and Skelton 2008). Until recently, water washing is the common method for biodiesel purification, but the method uses large quantities of water. Significantly, water use produces a massive amount of polluted effluent. The contaminated wastewater needs to be treated before it is released to the environment due to high pH values, high biological oxygen demand (BOD), and chemical oxygen demand (COD) contents (Wall et al. 2011; Atadashi 2015). Consequently, a large amount of water consumption is not feasible in treating the produced wastewater (Samsuri et al. 2017).

One of the alternatives to replace the water washing process is by using the dry washing method. This method uses solid adsorbents or ion exchange resins to purify biodiesel. Although it can replace the water washing process, the knowledge of its chemistry and the regeneration of spent adsorbent is still lacking (Atadashi 2015). Besides, a filter is needed to increase the effectiveness of this method in purifying biodiesel. Some researchers have been developing a membrane technology system to purify biodiesel (Torres et al. 2017; Kusworo et al. 2020). Many efforts have been made in controlling membrane fouling. However, the membrane fouling process causes the membrane to have low membrane flux and permeability, which increases the cleaning chemical costs, energy demand, and operating cost of the membrane system and shortens the lifetime of the membrane. Therefore, solvent-aided crystallization (SAC) is introduced to replace the water washing to minimize the difficulties encountered in biodiesel purification.

In this study, SAC was used to purify the biodiesel. SAC is a crystallization method that uses additional assisting agents (e.g. solvents). The use of assisting agent assists and affects the crystallization kinetics favorably. Although this type of crystallization can produce high-purity products, the viscosity of the melt has a negative effect on separation efficiency and the growth rates of the crystal (Eisenbart et al. 2017). The viscous product has difficulty with the nucleation process, growth, and post-treatment process. It will slow the diffusion rate and natural convection and subsequently develop the impure melt layer. Besides, the conventional melt layer crystallization is only suitable for a sample with lower viscosity. The sample used in this study is high in viscosity (i.e. biodiesel and glycerol) (Binhayeeding et al. 2017). In addition, researchers used liquid ammonia and 1-butanol as the additional assisting agents to purify glycerol via crystallization. As a result, both solvents can enhance crystal formation in suspension crystallization, but a 1-butanol solution can decrease the viscosity without affecting the vapor pressure of crystallization substances (Hass and Patterson 1941).

Recent studies have reported that the addition of 1-butanol has altered the kinetics of crystallization by lowering glycerol viscosity, improving melt agitation while stirring and enhancing purification with a high crystal growth rate (Thongboonkerd et al. 2006; Eisenbart and Ulrich 2015). The studies also proved that 1-butanol is the most suitable solvent, which significantly impacts the separation of glycerol and water; therefore, SAC is seen as a feasible glycerol method purification. On top of that, the traces of 1-butanol can be easily removed completely via evaporation from the mixtures because 1-butanol is discovered to remain largely in the liquid phase (Eisenbart and Ulrich 2016). The purification method via SAC was conducted right after the transesterification process in previous literature, which replaced gravitational settling and water washing (Singh 2011). As a result, high purity of biodiesel (% FAME purity) was obtained, which confirmed the capability of SAC in removing the contaminants that exist in the biodiesel. Nevertheless, the information of free glycerol content in purified biodiesel is not mentioned, but the higher the FAME purity percentage means, the lesser the contaminants present in the biodiesel and the limitation of the glycerol content (Singh 2011).

Therefore, this study attempts to remove free glycerol from FAME using the SAC method. The SAC method was done right after removing glycerol and contaminants by gravitational settling. The remaining free glycerol and other impurities left in the biodiesel were then removed using this SAC method. The composition of FAME in biodiesel and the total content of free glycerol were determined by using gas chromatography–mass spectrometry (GC–MS). Consequently, by measuring the purity of FAME, the percentage of glycerol and another contaminant such as K or Na also can be known. In addition, thermal analysis using differential scanning calorimetry (DSC) and rheometer was also used to analyze biodiesel and glycerol characteristics.

## Materials and methods

### Materials

Cooking oil from Buruh brand was purchased from a local market. Methanol (99.97% purity) and potassium hydroxide (KOH) pellets were supplied by Avantis Laboratory Supply. Meanwhile, ethylene glycol and 1-butanol were provided by Benua Sains Sdn. Bhd. Ethylene glycol solution of 50% (v/v) with water was used as the coolant inside the refrigerated bath.

### Synthesis of biodiesel via transesterification method

**Fig. 1:**
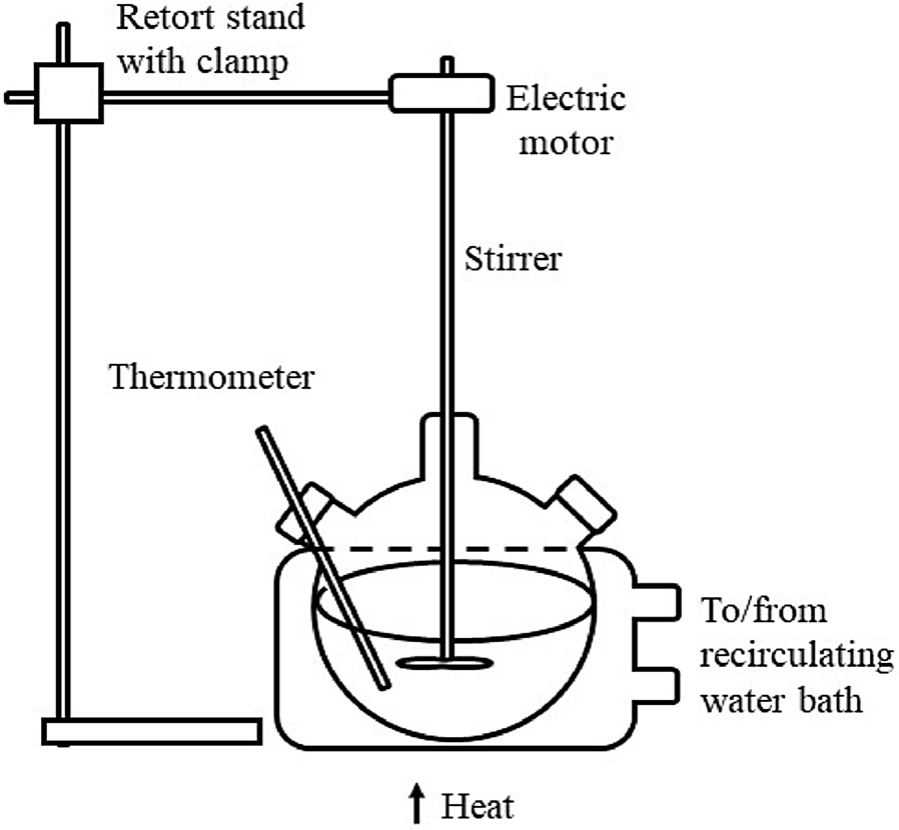
Transesterification reactor setup for biodiesel production

Figure 1 shows the experimental setup of the transesterification method. About 460 g of palm oil from Buruh brand was heated in the three-necked flask up to 60 °C. The temperature of palm oil was maintained between 55 and 60 °C using a recirculating water bath facility. The stirring speed and temperature were controlled by heating and stirring the mantle. About 3.5 g of KOH was measured using an enclosed electronic measuring cylinder and dissolved in 79.2 g of methanol. Then, the methoxide solution was added to the heated palm oil, and the reaction mixture was stirred rapidly for 20 min at 500 rpm using a magnetic stirrer. Then, the crude biodiesel was transferred into a separatory funnel and left for one day. After the gravitational settling process, the bottom glycerol layer was drained using a stop cock, and the volume of glycerol was recorded. The same step was repeated by draining the remaining biodiesel layer, and the volume was recorded.

### Glycerol removal via solvent-aided crystallization

**Fig. 2:**
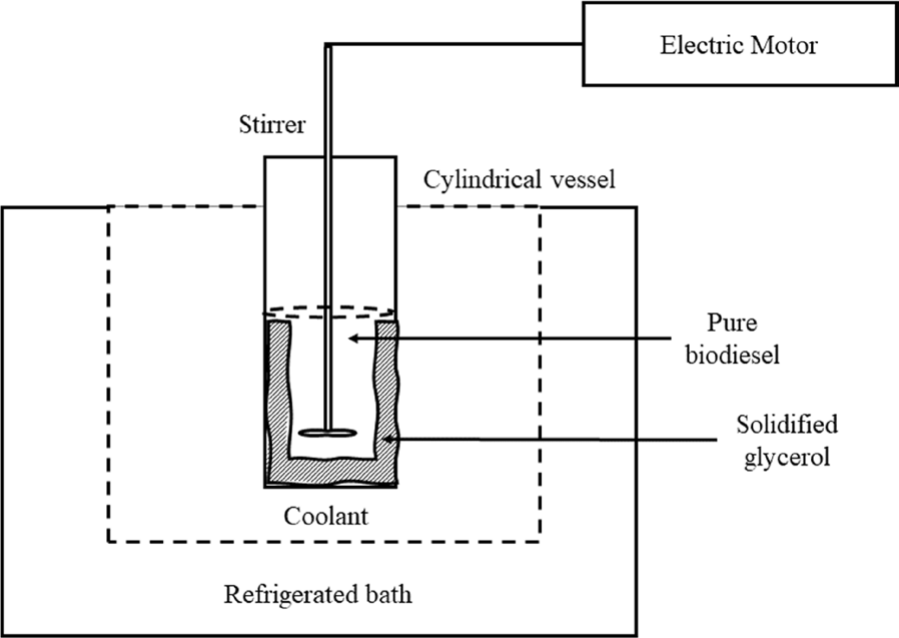
SAC system setup

Figure 2 shows the experimental apparatus setup for the SAC system using a cylindrical vessel (13.5 × 17 cm). A stainless-steel cylindrical vessel was used for solvent added with crystallizer to avoid the vessel from corroding and minimize the presence of foreign compounds in the solution (Samsuri et al. 2014). The vessel was equipped with a stirrer (EURO-ST 40 D S002, IKA, Malaysia) to stir the crude biodiesel at the solid–liquid interface. This is to allow the user to set the torque and speed for consistent mixing of the solution. Besides, it is to enhance the separation of glycerol as the FAME movement at solid–liquid was well promoted. A refrigerated bath (EYELA Cool ACE CA-1111, TOKYO RIKAKIKAI CO., LTD) was used to maintain the desired temperature sample. Then, the refrigerated bath was turned on, and the temperature was set at 8 °C.

**Fig. 3:**
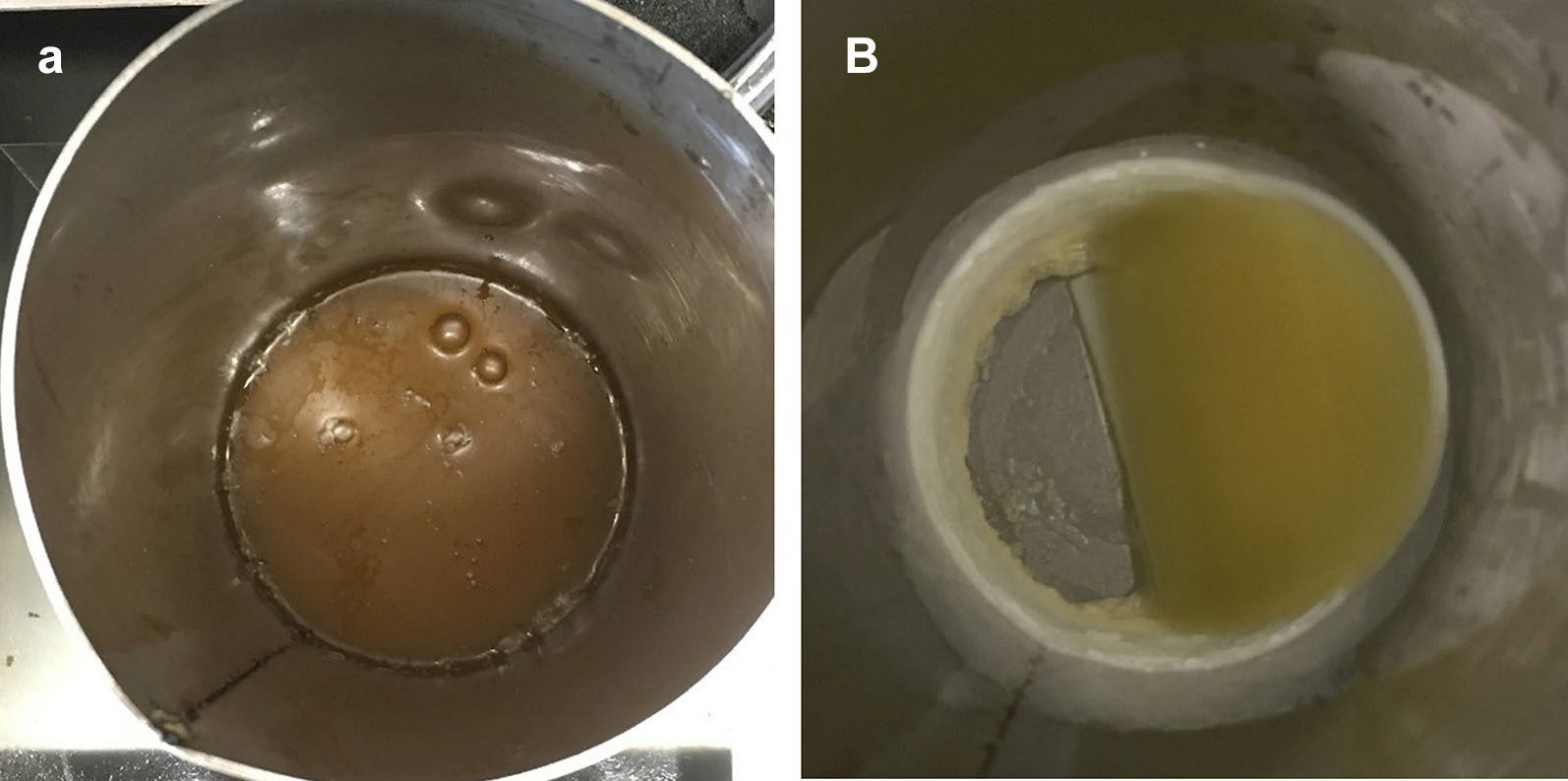
**a** Sample solution before SAC method and **b** solidified free glycerol attached on vessel surface, and pure biodiesel remained in liquid phase after SAC

A 500 ml volume of crude biodiesel and 1-butanol solvent with 1 wt. % of concentration was poured into the attached cylindrical vessel inside the refrigerated bath. Then, the stirrer was turned on, and the crystallization time was set for 35 min. Afterwards, stirring was stopped at the designated time, and the vessel was taken out from the refrigerated bath. Two phases of the sample were observed: the solid phase (the glycerol and other impurities) and the liquid phase (the pure biodiesel). The purified biodiesel was collected for further analysis using GC–MS. The experiment was repeated using different operating conditions such as different coolant temperatures (10, 12, 14, and 16 °C), stirring speed (100, 200, 400, and 500 rpm), and cooling time (20, 25, 30, and 40 min). All the biodiesel samples were evaluated using GC–MS to determine the FAME content as the purity of biodiesel. As shown in Fig. 3a before the purification via the SAC method and Fig. 3b the pure biodiesel remained in the liquid phase, and the solidified free glycerol and other contaminant attached to the vessel surface after SAC.

### GC–MS analysis

The GC–MS Shimadzu (GC–MS QP 2020, Shimadzu, Kyoto, Japan) was used to determine the total FAME content in the biodiesel. A column with an internal diameter of 30 mm and a film thickness of 0.25 µm from SGE and BP-20 (WAX)-polyethylene glycol was used. The chromatogram peaks were generated using the GC–MS solution equipped with 250 °C of injector temperature, 23 °C/min of oven temperature, and increase to 250 °C and 200 °C of ion source temperature. Helium gas was also used as the carrier gas, while *n*-heptane was used as the diluent. The purity of each process of purification was calculated using Eq. (1):

1 $$\% ~{\text{Composition~of~FAME}} = ~\frac{{{\text{Peak~are~of~individual~component}}}}{{{\text{Sum~of~correction~area}}}}~ \times 100\% ~$$

### DSC analysis

To determine crystallization temperature, both biodiesel and glycerol samples were analyzed using DSC Q2000 (TA Instrument-Waters, LLC, USA). The thermograms for both samples' heat flow were generated using the Q series (Q2000-2580-DSCQ2000) software. Biodiesel was calibrated to 30 °C and cooled to − 15 °C at a rate of 5 °C/min. Meanwhile, the temperature range for the glycerol sample was 0 to 30 °C at the same heating rate of 5 °C/min. DSC analysis is essential to determine biodiesel and glycerol's crystallization temperatures so that the suitable cooling temperature range can be used during purification via SAC.

### Rheometer analysis

Discovery Hybrid Rheometer, DHR-1 (TA Instrument, Elstree, UK)) rotational rheometer with a single wall rotor and the concentric cylinder was used to determine the rheological properties such as viscosity, shear stress, and a shear rate of biodiesel and glycerol. Besides, TA Instrument Trios version 4 software with a flow temperature ramping method. The purpose of this analysis was to justify that the viscosities of biodiesel and glycerol are higher than 0.1 Pa∙s. Therefore, this purification method via SAC can be used or suitable for highly viscous liquids sample (Eisenbart and Ulrich 2016).

### Statistical analysis

The analysis of variance (ANOVA) was utilized to find the significant difference error in this research by using STATISTICA software Version 8.0 Inc., USA. The experiment was conducted with a stirring speed of 100–500 RPM, a coolant temperature of 8–16 °C, and a crystallization period of 20–40 min. The experiment was repeated three times, with the average values determined. The correlation coefficient (*R*^2^) was used to assess the accuracy of the curves, and a value 0.05 of p-value was considered significant.

### Energy analysis

An amount of sample (biodiesel) was employed in this lab-sized experiment as the energy consumption of the biodiesel is determined by SAC method. The SAC's energy consumption is concentrated on the cooling energy (refrigerated bath) and the stirring energy (propeller). As the system carries out the operation, both types of equipment employ the same equation for specific energy consumption (kWh/m^3^). The specific energy consumption was calculated using Eq. (2), and (3) was used to compute the digital stirrer’s power (Power-Torque. 2002; Samsuri et al. 2020):

2 $${\text{SEC}}_{{{\text{equipment}}}} = \frac{{P_{{{\text{equipment}}}} \times \eta _{{{\text{equipment}}}} }}{{V_{{{\text{sample}}}} }}$$

3 $$P_{{{\text{propeller}}}} = \frac{{T \times N_{{{\text{rpm}}}} }}{{9548.8}}.$$

The power of equipment (kW) indicates as P, followed by η is the efficiency of the equipment, which has been set at the minimal point (80%) for both equipment. V is the volumetric flowrate (m^3^/h) in Eq. (2). Next, in Eq. (3) T indicates the torque of the propeller (0.4 Nm), and *N*_rpm_ referred the speed of the propeller for each run of experiment. The overall specific energy consumption is shown in Eq. (4), which is the sum of the refrigerated bath and propeller's specific energy consumption:

4 $${\text{SEC}}_{{{\text{Total}}}} = {\text{SEC}}_{{{\text{refrigerated~bath}}}} + {\text{SEC}}_{{{\text{propeller}}}}$$

## Results and discussion

### Biodiesel analysis by GC–MS

**Fig. 4:**
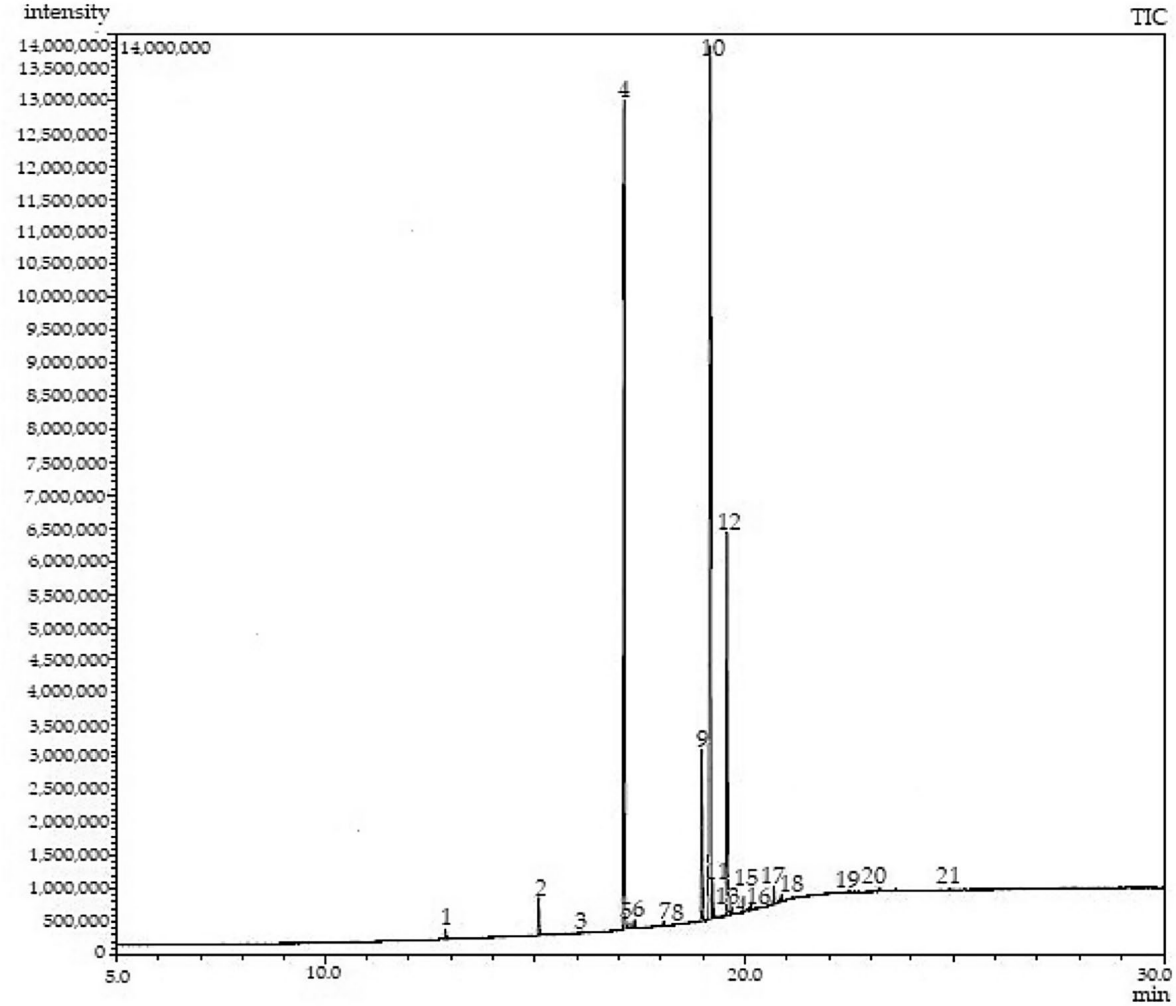
GC–MS chromatogram of crude biodiesel

The purity of FAME content after the gravitational settling process was identified using the GC–MS. The chromatogram of crude biodiesel with a graph of intensity versus retention time is plotted in Fig. 4, followed by the peak data in Table 1 to determine the systematic names of fatty acids found in the palm oil-based crude biodiesel.

**Table 1:** GC–MS results for crude biodiesel separated from glycerol after gravitational settling

Peak number	Retention time, t_R_	Library/ID (systematic name)	Trivial name	Types of fatty acids	Composition of FAME% (purity)
1	12.870	Dodecanoic acid	Lauric	Saturated	0.31
2	15.092	Tetradecanoic acid	Myristic	Saturated	1.22
4	17.128	Hexadecanoic acid	Palmitic	Saturated	35.08
6	17.375	9-Hexadecenoic acid	Palmitoleic	Unsaturated	0.25
9	18.973	Octadecanoic acid	Stearic	Saturated	6.03
10	19.173	9-Octadecenoic acid	Oleic	Unsaturated	40.54
12	19.572	9,12-Octadecadienoic acid	Linoleic	Unsaturated	14.41
15	20.13	9,12,15-Octadecatrienoic acid	Linolenic	Unsaturated	0.23
17	20.684	Eicosanoic acid	Arachidic	Saturated	0.61
	Total				98.96%

Table 1 shows the peak number, systematic name (Library/ID), trivial name, and the percentage of FAME composition in palm oil-based biodiesel. According to the Palm Oil Research Institute of Malaysia (PORIM), the type of fatty acids present in palm oil are lauric, myristic, palmitic, palmitoleic, stearic, oleic, linoleic, linolenic, and arachidic. Based on Table 1, the total percentage composition of FAME or biodiesel produced from the gravitational settling method was 98.96%.

In Table 1, the total amount of saturated fatty acid and unsaturated fatty acid was 43.25% and 55.71%, respectively. The percentage values mostly are between the range specification of biodiesel by PORIM as in Table 2 (Crabbe et al. 2001).

**Table 2:** PORIM specification of biodiesel composition (Crabbe et al. 2001)

Parameters	PORIM specification (%)
Lauric	0.0–0.4
Myristic	0.6–1.6
Palmitic	41–47
Palmitoleic	0–0.6
Stearic	3.7–5.6
Oleic	38.2–43.5
Linoleic	6.6–11.9
Linolenic	0.0–0.5
Arachidic	0.0–0.8
Unsaturated fatty acid	44.8–57.3
Saturated fatty acid	45.3–44.5

The info such as correction area of individual component and sum of correction area is provided from the table. Thus, the percentage composition of individual FAME can be calculated using Eq. (1). Both data can be obtained from Table 3.

**Table 3:** GC–MS peak data

Peak	Retention time	Area
1	12.870	240,375
2	15.092	945,990
3	16.125	38,770
4	17.128	27,201,103
5	17.311	23,262
6	17.375	193,851
7	18.06	46,524
8	18.279	23,262
9	18.973	4,675,674
10	19.173	31,434,798
11	19.224	550,535
12	19.572	11,173,543
13	19.675	116,310
14	20.072	23,262
15	20.13	178,342
16	20.242	23,262
17	20.684	472,995
18	20.866	62,032
19	22.466	31,016
20	23.192	46,524
21	24.916	38,770
	Total	77,540,202

For Peak 3, lauric:

$${\text{\% Composition~of~FAME~}} = {\text{~}}\frac{{240375}}{{77540202}} \times 100\% = 0.31\%$$

### DSC analysis

**Fig. 5:**
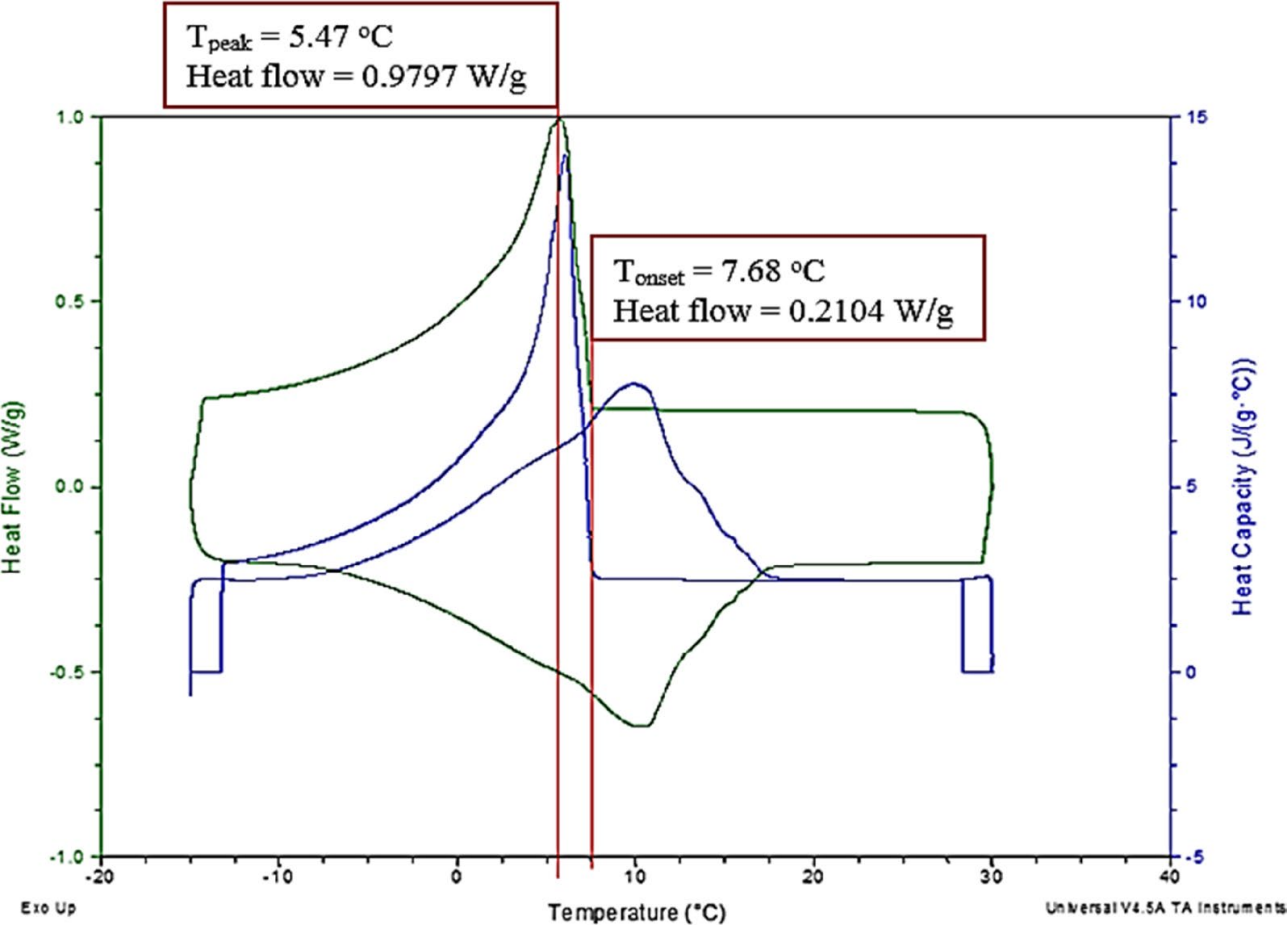
DSC thermogram for crude biodiesel

**Fig. 6:**
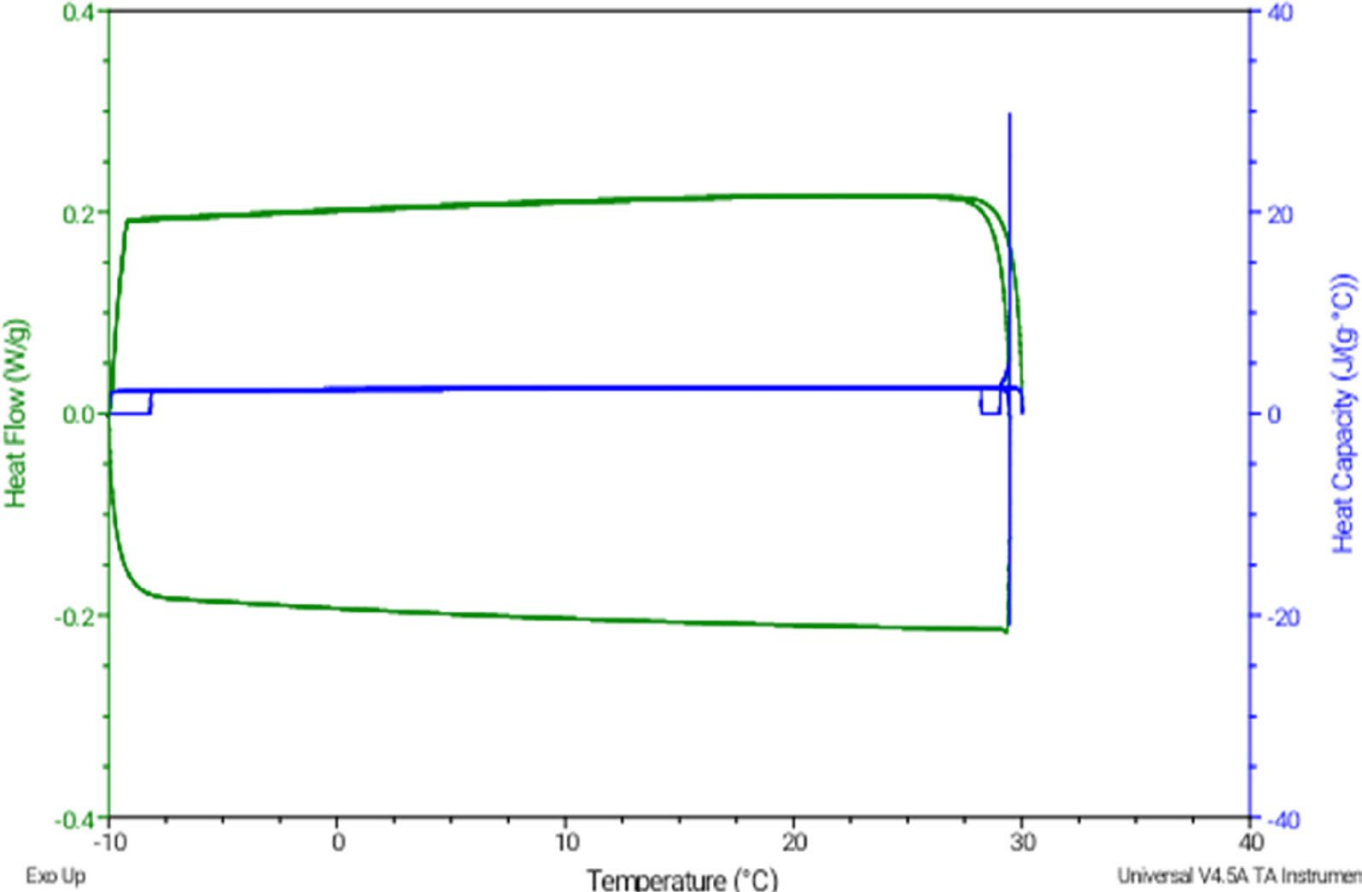
DSC thermogram for crude glycerol

Figures 5 and 6 show the thermogram of biodiesel and glycerol. The green line indicated heat flow, and the blue line indicated heat capacity, respectively. The analysis was done for the biodiesel sample which was cooled from 30 to − 15 °C at a constant rate of 5 °C/min (green line) in Fig. 5. The appearance of the exothermic peak was due to the heat flow out of the sample because the sample is cooling specifically during crystallization. Crystallization consists of a two-step process, which is nucleation and solid growth (Çaylı and Küsefoğlu 2008). The graph shows that the onset temperature was 7.68 °C, indicating that the crystallization of the biodiesel started to occur. Then, it reached 5.47 °C show the maximum or peaks as the maximum crystallization temperature of biodiesel.

On the contrary, no visible peaks were observed in the thermogram for the glycerol sample in Fig. 6. The cooling rate for biodiesel was also used on glycerol with a different temperature range (30 to − 20 °C) and fixed heat flow. The result is also supported by a recent study on the cold properties of fuel mixture and crude glycerol using the DSC analysis, which reported that glycerol has a melting point of 17.8 °C, and no peak was observed during the DSC analysis due to the occurrence of supercooling (Gao et al. 2017).

### Rheological properties

**Fig. 7:**
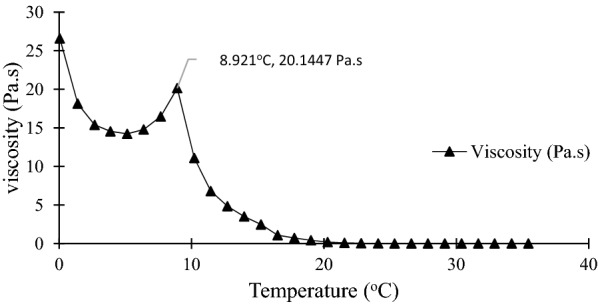
Viscosity curve of biodiesel

Figure 7 shows that the viscosity of biodiesel at 8.92 °C was 20.1 Pa∙s. The biodiesel was cooled to − 10 °C and heated up to 35 °C.

**Fig. 8:**
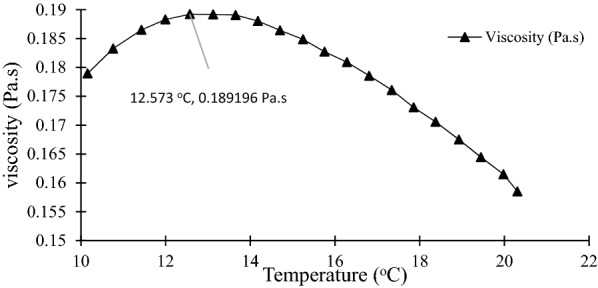
Viscosity curve of glycerol

Meanwhile, Fig. 8 shows that the viscosity of glycerol at 12.6 °C was 0.189 Pa∙s, which was heated from 10 to 20 °C. Biodiesel and glycerol viscosity decreased as temperature increased (Björn et al. 2018; Silva et al. 2015). This is because as temperature rises, fluid flow in a system is increased. Thus, it is proven that the SAC process can be used for highly viscous liquid irrespective of the impact of temperature on viscosity as the viscosity of biodiesel and glycerol was higher than 0.1 Pa∙s (Eisenbart and Ulrich 2016).

### Effect of coolant temperature on SAC

**Fig. 9:**
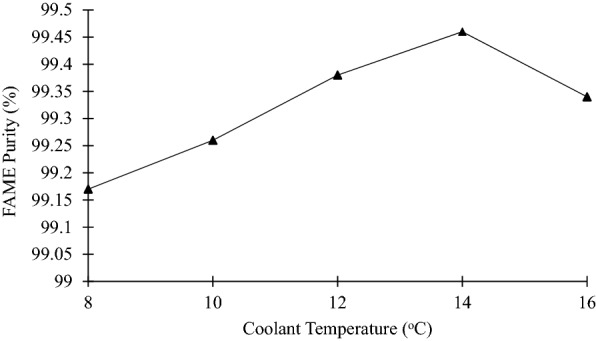
Graph of FAME Purity against coolant temperature

Figure 9 shows that the FAME purity increased when the temperature increased from 8 to 14 °C, and this increase is statistically significant (*p* < 0.05) and show an *R*^2^ value of 0.89. The lowest percentage of 99.17% at 8 °C was due to the coolant temperature, which is very close to the crystallization temperature of biodiesel as mentioned in Fig. 5. As a result, the lowest FAME purity was obtained by solidifying certain fatty acids due to the slight differences range of temperature between coolant and crystallization temperature of biodiesel.

In addition, the rate of ice crystals or solid growth is controlled by coolant temperature (Amran and Jusoh 2016; Jusoh et al. 2014a). When the temperature is close to the crystallization point, the likelihood of FAME being trapped is higher within the solid layer formed by glycerol and other impurities. Under lower coolant temperature, solid growth rate tends to be greater, which induced a higher methyl ester incorporation into contaminants solid (Samsuri et al. 2017). In addition, the thickness of the solid formed increases as the temperature of the coolant reduces. Nevertheless, the formation of a thicker solid led to high product purity (Mohamad et al. 2017). Hence, the coolant temperature influences the purity of FAME produced.

In contrast, the highest purity of FAME was achieved at 99.46% at 14 °C, because this temperature is considered as the favorable coolant temperature. In this state, the FAME did not form in the solid phase and stayed in the liquid phase, while other impurities, including glycerol, were attached to the wall. As observed, solid growth was in an ordered pattern as temperature rises (Romli et al. 2009). In fact, at a higher coolant temperature, the solid can grow in the ordered pattern that enables the pure FAME to free from contaminants and accumulate in the solution and elevates the purity of FAME (Samsuri et al. 2017). However, too high of coolant temperature can affect the solid formation. At higher temperature, the inclusion of solutes can melt and dilute into the solution (Bagdasarov 1988). As a result, the percentage of FAME decreased to 99.34% at 16 °C. Thus, the coolant temperature of 14 °C is considered the ideal temperature to obtain high-purity FAME (FAME yield = 99.32 ± 0.11%).

### Effect of stirring rate on SAC

**Fig. 10:**
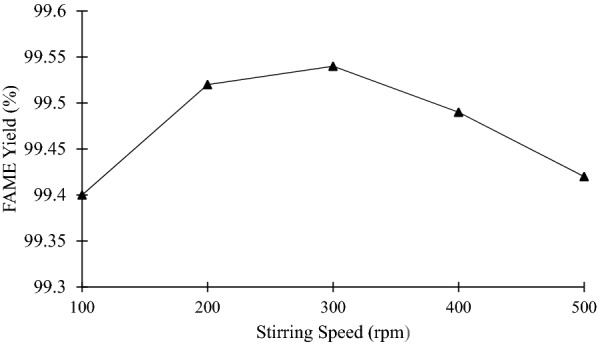
Graph of FAME purity against stirring speed

Solution movement assistance is crucial in enhancing the solid to be formed. A propeller was used to perform this SAC system. Besides, a gentle motion is required to maintain the uniform distribution of temperature and the flow of the system (Mohamad et al. 2017). In Fig. 10, an increasing trend observed from (100 to 300 rpm) and decreases gradually at 400 and 500 rpm.

Accordingly, 300 rpm of stirring rate produced a higher percentage of FAME purity (99.54%) due to the suitable agitation speed to diminish the build-up or accumulation of solute near the liquid–solid interface (Ab. Hamid et al. 2015). Flowrate is closely associated with the circulation of fluid, whereby an increase in flowrate will eventually decrease the advanced rate of the solid front (Miyawaki et al. 2005). As a result, the increase in FAME percentage indicates a better separation efficiency from glycerol and other contaminants. Furthermore, a high circulation flowrate also imposes the development of a high shear force, which can separate the solute from the solution (Jusoh et al. 2014b). High shear force due to high flowrate enables the contaminants solid formed to be carried away easily from the solution, leaving the biodiesel with a higher percentage of FAME. On top of that, the FAME purity was the lowest at 100 rpm (99.40%). A slower stirring speed induces slower movement of solutions and reduces the efficiency of separation. Thus, it explains that mild agitation is preferred compared to slower stirring speed.

For the stirring rate of 400 and 500 rpm, the reduction of FAME purity was 99.49% and 99.42%, respectively. The FAME yield was significant (*p* < 0.05) with the *R*^2^ value of 0.96. This is because the higher stirring speed can erode the solid formed onto the vessel wall (Romli et al. 2009). The turbulence flow was created inside the system when the agitation was more heightened. Thus, the solidified contaminant was mixed with the biodiesel, eventually reducing the FAME purity. Besides, vigorous stirring will promote a slower solidification rate and reduce the liquid phase's final concentration (Mohamad et al. 2017; Ab. Hamid et al. 2015). Consequently, the contaminants crystallization rate is slowed down, limiting the heat transfer, and finally decreases the final concentration of FAME (FAME yield = 99.47 ± 0.06%).

### Effect of crystallization time on SAC

**Fig. 11:**
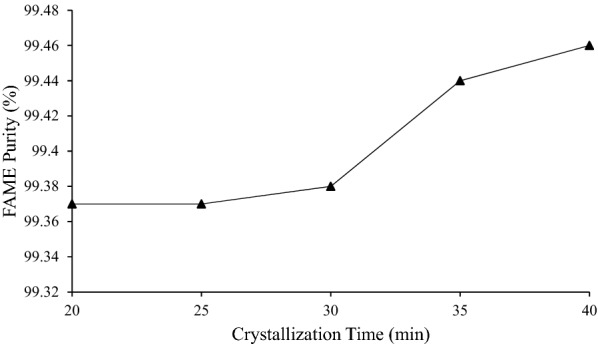
Graph of FAME purity against crystallization time

The plot (*p* < 0.05) in Fig. 11 shows that FAME purity increased when the temperature increased up to 99.46% (*R*^2^ = 0.93). This is because longer crystallization time enhances the crystallization process. When a solution is stirred for adequate time, it provides longer residence time to be in the crystallizer and allows thicker solid layer formation (Jusoh et al. 2014b). Thus, more contaminants were trapped within the thick solid layer and left the unfrozen biodiesel with higher FAME purity. At 20 and 25 min, the FAME purity remained unchanged with 99.37%, indicating that the crystallization process just started to occur (FAME yield = 99.40 ± 0.04%). Therefore, sufficient cooling time is needed for better purity of biodiesel. However, a crystallization time longer than 40 min was not conducted further in this study.

In the F-test, the coefficients are added to the system and being analyzed. The experiment results might lead to improvements in system parameters and make the system better match in the experiment. The calculated F-value was 4.84, as shown in Table 4, which is greater than the tabulated *F*-value of 95% confidence (*F*_0.05_, _3_, _11_) which is 3.587 thus, that the regression model as a whole is statistically significant.

**Table 4:** ANOVA result for regression

Source	Sum of squares	Degree of freedom	Mean squares	*F*-value
Regression	0.074	3	0.0246	4.847
Residual	0.056	11	0.0050	
Total	0.130	14		
*R*^2^	0.569			

### Energy consumption

Nowadays, the most challenging part for the industry is reducing energy consumption to reduce operational costs (Innocenzi and Prisciandaro 2021). The energy consumption for SAC method was calculated using the power of equipment measured through the term of the energy used for the contaminant removal. The energy consumption was varied for each run by the formation of the solid. The highest total energy consumption 323.0072360 kWh/m^3^ was achieved due to the longer time of the experiment conducted.

The optimum parameter for total energy consumption is the highest number of energies consumed. Thus, based on the analysis of the energy consumption of contaminant removal at the lower crystallization time 161.5036180 kWh/m^3^ of energy consumption was achieved. This shows that the crystallization time was the crucial parameter to be considered. To conclude that, to compare both results, the lower energy value should be taken into account as both FAME purity show almost similar numbers (Table 5).

**Table 5:** Calculation of energy consumption

Run	Stirring rate (RPM)	Time (h)	FAME purity (%)	Volume of sample (m^3^)	Volumetric flowrate (m^3^/h)	Energy of propeller (kWh/m^3^)	Energy refrigerated (kWh/m^3^)	Total energy (kWh/m^3^)
1	300	0.583	99.17	0.0005	0.000857	2.9311	279.82	282.7525805
2	300	0.583	99.26	0.0005	0.000857	2.9311	279.82	282.7525805
3	300	0.583	99.38	0.0005	0.000857	2.9311	279.82	282.7525805
4	300	0.583	99.46	0.0005	0.000857	2.9311	279.82	282.7525805
5	300	0.583	99.34	0.0005	0.000857	2.9311	279.82	282.7525805
6	100	0.583	99.40	0.0005	0.000857	0.9768	279.82	280.7982609
7	200	0.583	99.52	0.0005	0.000857	1.9536	279.82	281.7750709
8	300	0.583	99.54	0.0005	0.000857	2.9311	279.82	282.7525805
9	400	0.583	99.49	0.0005	0.000857	3.9058	279.82	283.7272919
10	500	0.583	99.42	0.0005	0.000857	4.8828	279.82	284.7043351
11	300	0.333	99.37	0.0005	0.001501	1.6742	159.82	161.5036180
12	300	0.416	99.37	0.0005	0.001201	2.0915	199.66	201.7582736
13	300	0.500	99.38	0.0005	0.001000	2.5138	239.98	242.4979250
14	300	0.583	99.44	0.0005	0.000857	2.9311	279.82	282.7525805
15	300	0.666	99.46	0.0005	0.000750	3.3484	319.65	323.0072360

## Conclusion

The SAC system has proven to remove contaminants from crude biodiesel, which is applicable as a waterless method for biodiesel purification. This study aims to determine the effectiveness of free glycerol from FAME using the SAC method. The highest FAME purity and the lower energy consumption removal of contaminants from biodiesel was 99.37% and 161.5036180 kWh/m^3^. Therefore, SAC must be operated at a coolant temperature of 14 °C, stirring rate of 300 rpm, and crystallization time of 20 min. It is essential to work with precision to form pure contaminants solid and recover the biodiesel. Thus, the increase of final FAME content in biodiesel indicates the presence of impurities is limited.

## Data Availability

All data generated or analyzed during this study are included in this published article [and its additional information files].
